# The accuracy of navigated versus freehand curettage in bone tumors: a cadaveric model study

**DOI:** 10.1007/s11548-022-02741-w

**Published:** 2022-11-03

**Authors:** Thomas R. F. van Steenbergen, Han Nijsink, Thomas G. E. Eggen, Dennis Janssen, Maroeska M. Rovers, Ingrid C. M. van der Geest, J. J. Fütterer

**Affiliations:** 1 Department of Medical Imaging, Radboudumc, P.O. Box 9101, Nijmegen, 6500 HB The Netherlands; 2 Department of Orthopaedics, Radboudumc, Nijmegen, The Netherlands; 3 Department of Operating Rooms, Radboudumc, Nijmegen, The Netherlands

**Keywords:** Computer-assisted surgery, Navigation, Bone, Tumor, Orthopedic, Curettage

## Abstract

**Purpose:**

Navigation has been suggested to guide complex benign bone tumor curettage procedures, but the contribution of navigation to the accuracy of curettage has never been quantified. We explored the accuracy of navigated curettage in a cadaveric observational pilot study, comparing navigated to freehand curettage, performed independently by an expert and a novice user.

**Methods:**

The expert performed curettage on 20 cadaveric bones prepared with a paraffin wax mixture tumor, 10 freehand and 10 navigated. We re-used 12 bones for the novice experiments, 6 freehand and 6 navigated. Tumor and curettage cavity volumes were segmented on pre- and post-cone-beam CT scans. Accuracy was quantified using the Dice Similarity Coefficient (DSC), and with remaining tumor volume, bone curettage volume, maximal remaining width and procedure times compared between navigation and freehand groups for both users.

**Results:**

There were little differences in curettage accuracy between a navigated (DSC 0.59[0.17]) and freehand (DSC 0.64[0.10]) approach for an expert user, but there were for a novice user with DSC 0.67(0.14) and 0.83(0.06), respectively. All navigated and freehand procedures had some amount of remaining tumor, generally located in a few isolated spots with means of 2.2(2.6) cm^3^ (mean 20% of the tumor volume) and 1.5(1.4) cm^3^ (18%), respectively, for the expert and more diffusely spaced with means of 5.1(2.8) cm^3^ (33%) and 3.0(2.2) cm^3^ (17%), respectively, for the novice.

**Conclusions:**

In an explorative study on 20 cadaveric bone tumor models, navigated curettage in its current setup was not more accurate than freehand curettage. The amount of remaining tumor, however, confirms that curettage could be further improved. The novice user was less accurate using navigation than freehand, which could be explained by the learning curve. Furthermore, the expert used a different surgical approach than the novice, focusing more on removing the entire tumor than sparing surrounding bone.

## Introduction

Intralesional curettage is the preferred surgical treatment for most types of symptomatic benign bone tumors. The procedure is guided by anatomical information from preoperative imaging and by differences in tissue consistency between tumor and bone. Microscopic residual tumor is inherent to intralesional curettage of benign bone tumors, which has resulted in local recurrence rates of 20–65.2% [1]. Local adjuvant therapy is generally successfully employed to extend the surgical margin and reduce the tumor residue. The subsequent decrease in local recurrence rates depends on the type of adjuvant therapy [1]. Remaining local recurrences could potentially be prevented by improving adjuvant therapy, or by improving the accuracy of curettage.

If a tumor is situated in a challenging location, e.g., close to vital structures, surgeons need visualization of the procedure to control complete removal of the tumor tissue without compromising surrounding tissue. To achieve this, intraoperative imaging or image-guidance methods can be employed. Two-dimensional (2D) fluoroscopy is most commonly used, whereas surgical navigation offers three-dimensional (3D) guidance on preoperative or intraoperative 3D scans.

Surgical navigation has shown added benefit in bone sarcoma resections [2], with phantom and cadaver studies showing that the accuracy of navigated saw plane cuts was higher than freehand [3, 4]. Substantially less studies were performed on navigated benign bone tumor curettage, with conflicting results on its added benefit and difficult to compare studies as operating techniques and choice of adjuvants differed [5–7]. Furthermore, the question remains what the effect of navigation is on the accuracy of curettage, regardless of adjuvant therapy. More insights into its accuracy could help determine the role of navigation in the surgical treatment of benign bone tumors and could be useful for future guidelines. Additionally any beneficial effect of navigation might be larger for less experienced surgeons [3].

Therefore, in this study the accuracy of navigated curettage was compared to freehand curettage on a cadaveric bone tumor model. Secondly, the effect of navigation was compared between an experienced orthopedic surgeon (expert) and an orthopedic surgeon in training (novice).

## Methods

### Study design

We performed an observational pilot study, comparing navigated to freehand curettage, which was performed by both an expert and a novice user. First the expert performed curettage procedures on 20 cadaveric bone tumor models, of which 10 were done freehand and 10 navigated. The 12 bones that were re-usable after these initial expert experiments were again converted into bone tumor models. Second, a novice performed curettage procedures on these re-used models, which were also equally divided into a freehand (*n* = 6) and a navigated (*n* = 6) group.

### Cadaveric bone tumor model

The bone tumor models were created from 20 bare human cadaveric bones without surrounding soft tissue, as this was necessary to be able to implant the tumor phantom material. Furthermore, to test the accuracy of curettage only the bone itself was needed. Frozen (− 18 °C) proximal or distal femur or tibia segments were used as these contain curettable cancellous bone, in which tumor cavities could be created. The relative amount of each type of bone segment in this study was based on their prevalence in a sample of atypical cartilaginous tumor studies [8–10], which resulted in the inclusion of 5 proximal femurs, 11 distal femurs and 4 proximal tibias. The oncological approach for curettage in the femur and tibia is lateral, which is why the bones were bisected with a sagittal cut. Rounded cavities, elongated along one axis, were created at the same location in both bone halves. Cavity locations were varied in the craniocaudal and anteroposterior directions, as to create unique cases. There was no medial–lateral variation as all cavities were centered on the sagittal cut.

Bone tumor phantoms that mimic the consistency and curettability of a benign bone tumor were implanted in the cavities inside the cancellous bone, so that the surgeon could feel differences in tissue consistency between tumor and bone, representing clinical conditions. Three materials in various dilutions were tested for their suitability as a bone tumor phantom: silicone (Ecoflex Gel, Smooth-On, Macungie, PA), agar and paraffin wax, diluted with silicone thinner, water and mineral oil, respectively. Several small samples of each material in different dilutions were made. Two orthopedic surgeons compared the consistency and curettability of the samples to that of a benign cartilage tumor, based on their clinical experience. Phantom to bone contrast on cone-beam CT (CBCT) was also tested. Paraffin wax with mineral oil (1:2 dilution) was chosen for the experiments.

**Fig. 1:**
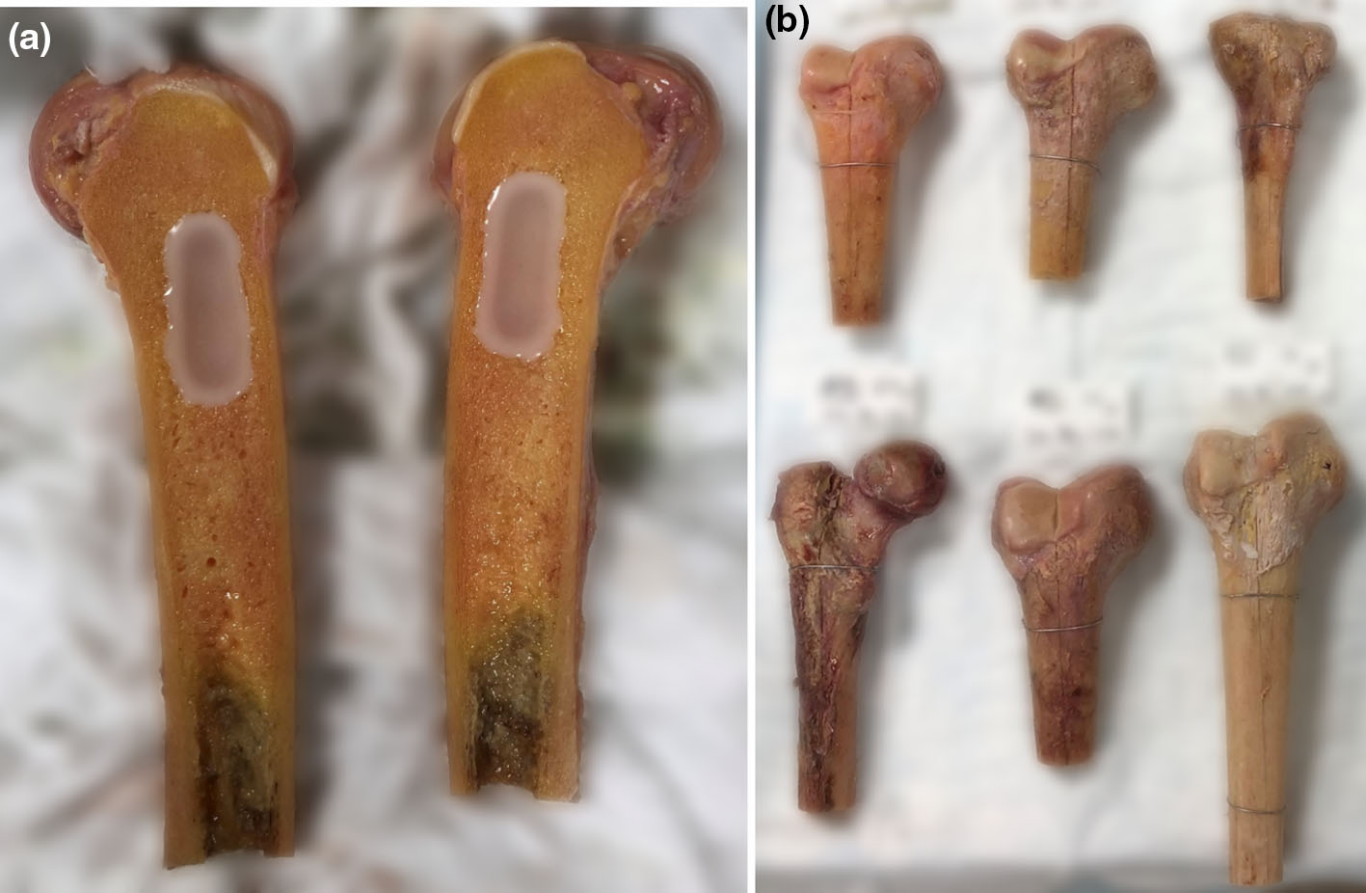
The cadaveric bone tumor model, with **a** being the situation just after filling the two cavities for bone 3. The paraffin wax mixture has started to solidify around the edges, but is still fluid in the center; **b** some examples of finished cadaveric bone tumor models, with bone 3 in the upper left corner

The tumor phantoms were implanted the day before the experiment to avoid re-freezing the final cadaveric bone tumor models. While the bones where thawing, the paraffin wax mixture was melted in a 70 °C oven and then poured in the cavities (Fig. 1a). After 5 min, some extra paraffin wax was added, as the mixture shrinks slightly after solidifying. Approximately 10 min after pouring the initial paraffin wax into the bone, the two halves were glued together again with an instant adhesive (LOCTITE 495, Henkel AG & Co. KGaA, Düsseldorf, Germany). The union was temporarily supported with cerclage at the diaphysis (Fig. 1b). Finally, each bone was imaged with a (CBCT) scan (Artis Zeego, Siemens Healthineers, Erlangen, Germany).

### Experimental setup

The bones were divided into two treatment groups: 1. navigated or 2. freehand curettage. Dependencies such as the type of bone, left or right sided bones, bone of the same donor, bone and tumor location were all taken into account as well as the distribution into groups was done manually. Treatment order was randomized in Excel (Microsoft Office Professional Plus 2016, Microsoft, Redmond, WA).

A Brainlab Curve (Brainlab, München, Germany) navigation system was used for the navigation group. The reference base was clamped to the bones, and the scans were registered by initial point-paired matching on anatomical landmarks, followed by surface matching using a cloud of 20 points. The registration accuracy was then visually verified using a tracked pointer. A registration accuracy of approximately 1 mm or less was accepted, as this is widely approved as a good registration accuracy [2].

**Fig. 2:**
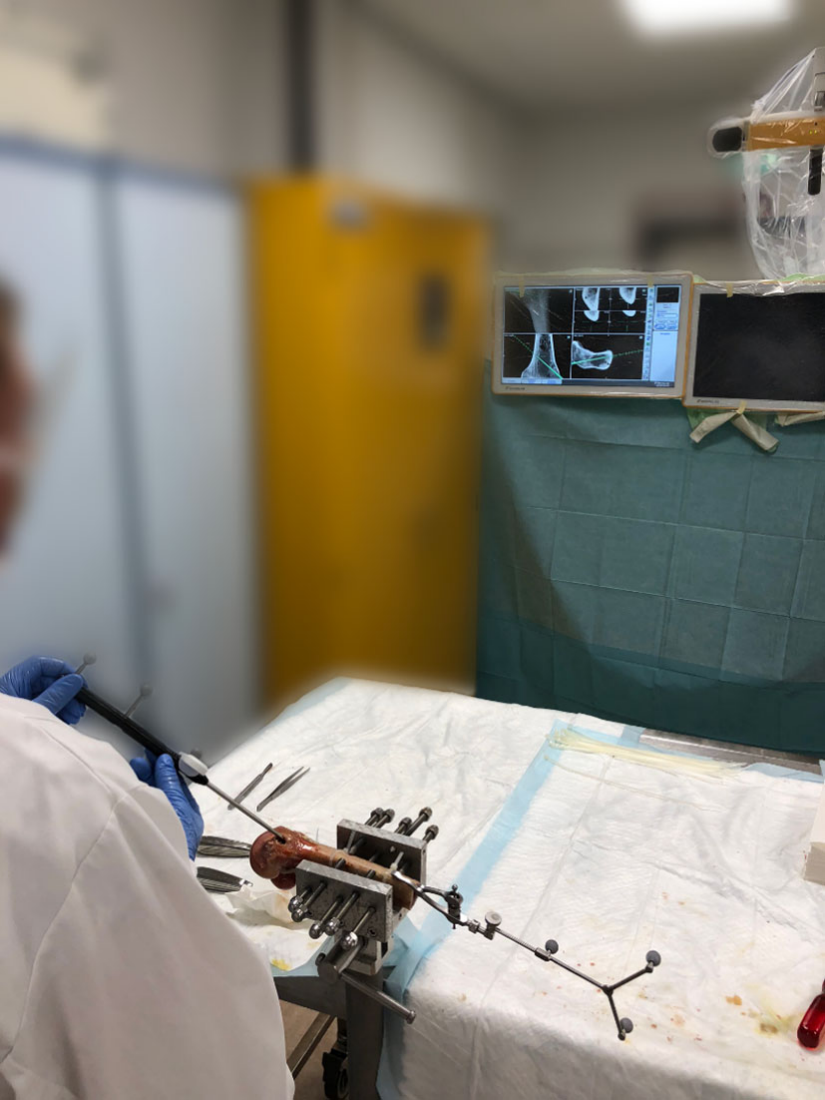
The experimental setup during navigated curettage of a distal femur. The surgeon uses the tracked pointer to assess the tumor shape and location

All experiments were performed in the orthopedic research laboratory of the Radboud University Medical Center (Nijmegen) (Fig. 2). Before each procedure, the performing orthopedic surgeon would plan the curettage by studying the CBCT. Meanwhile, a researcher clamped the bone to a table for fixation and registered the CBCT scan to the bone in the case of a navigated procedure. The goal of each procedure was to completely remove the tumor phantom, while removing as little healthy bone as reasonably possible. Available instruments included different sizes of curettes and a high-speed burr, supported by a tracked pointer and tracked curette for navigation cases. The procedure time, from picking up the first instrument to finishing curettage, was recorded for each procedure.

The navigation and freehand procedures were alternated, starting with an unrecorded testing procedure on a different bone for each group. For each user, the procedures were performed in a single day. At the end of the day, CBCT scans of the curetted bones were again acquired.

### Expert and novice

The initial 20 procedures were done by an expert user: an experienced orthopedic surgeon (IvdG, 11 years of experience in orthopedic oncology, 2 years of experience with navigation). The experiment was repeated three months later with a novice user: an orthopedic surgeon in training (TE, first year training for orthopedic surgeon, no experience with navigation). Bones that were still usable after the first experiment, i.e., had an intact and rounded cavity left by the curettage, were re-used for the second experiment. The bones were thawed, the paraffin wax mixture was poured into the curettage cavity through the bone window made during the first experiment, and after 10 min the bone window was sealed using fast-hardening denture base material (Autoplast, CANDULOR AG, Opfikon, Switzerland). The experimental setup was the same as in the first experiment except for the surgical approach, which was now medial for the distal femur and distal tibia, and lateral-distal (under the original approach) for the proximal femur.

### CBCT analysis

**Fig. 3:**
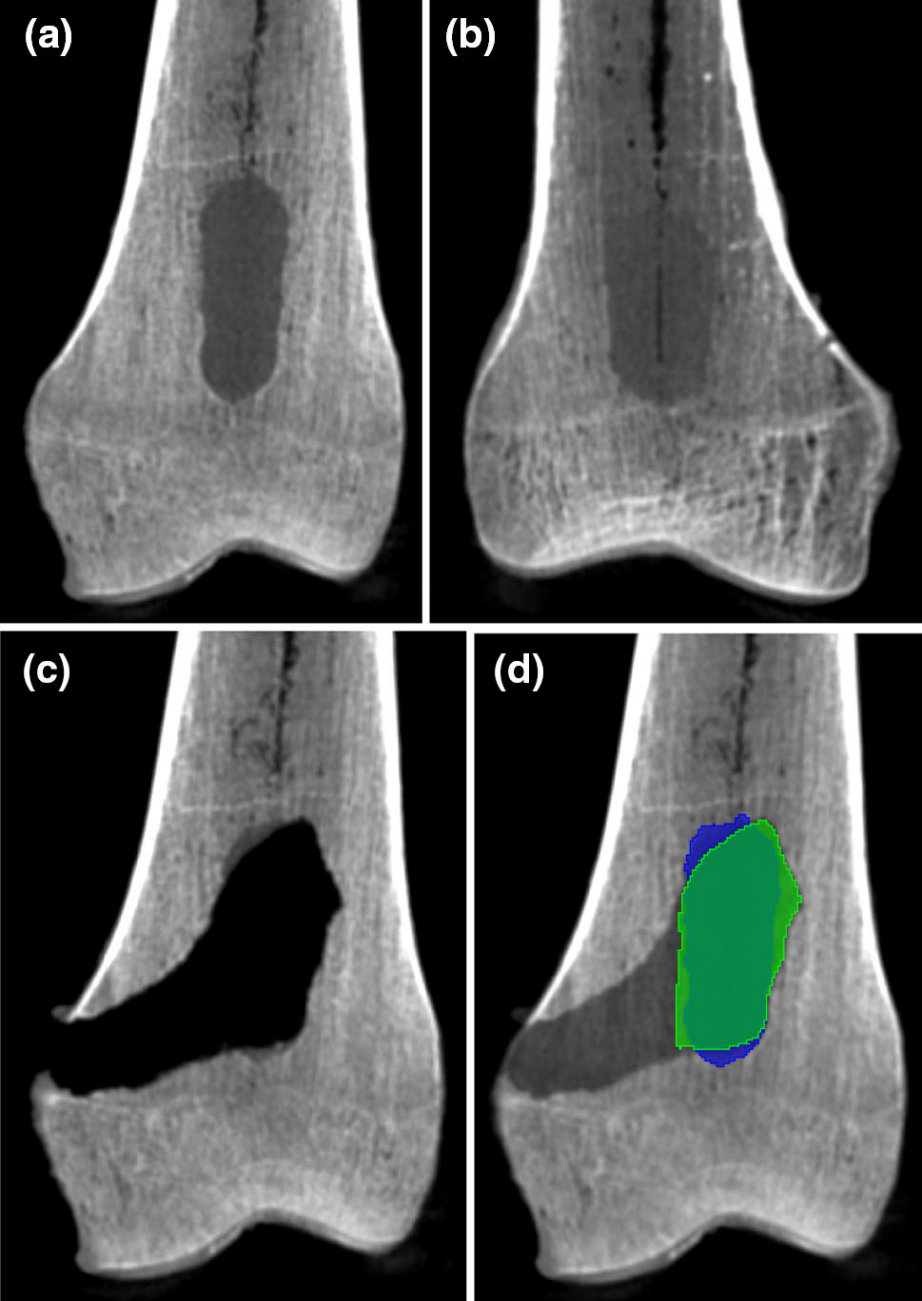
Cone-beam CT scans of the cadaveric bone tumor model; **a** model with good contrast between tumor and bone on the pre-CBCT (bone 3); **b** model with poor contrast between tumor and bone on the pre-CBCT; **c** the removed tissue for bone 3 on the post-CBCT, with tumor remaining at the proximal and distal ends; **d** the fused pre-CBCT (dark) and post-CBCT (light) of bone 3 with the segmentations of the tumor (blue) and curettage cavity (green), highlighting the remaining tumor parts

Each bone had a preprocedural CBCT (pre-CBCT) and postprocedural CBCT (post-CBCT) available (Fig. 3a–c) which were fused and segmented using the free and open source 3D slicer software (Fig. 3d; www.slicer.org) [11]. One researcher (TvS) performed all pre-CBCT and post-CBCT fusions manually and segmented the removed tissue from the post-CBCT scans based on a fixed gray value threshold. A second researcher (HN) checked these fusions (no visual misalignment between both scans) and segmentations (no holes and visually including the entire volume) for discrepancies that the first researcher would have to correct.

The two researchers segmented the tumor from the pre-CBCT independently based on a visual gray value threshold and manual refinements. The final tumor was constructed based on consensus using a Dice Similarity Coefficient (DSC): $DSC^{Tu} = \frac{{2\left| {Tumor1 \cap Tumor2} \right|}}{{\left| {Tumor1} \right| + \left| {Tumor2} \right|}}$. DSC is a dimensionless value calculated using both tumor segmentations. The volume of the intersect of both segmentations is multiplied by 2 and divided by the sum of both segmentation volumes; therefore, the DSC ranges from 0 to 1 with 1 meaning perfect similarity. Consensus was defined as DSC^Tu^ ≥ 0.95, in which case the final tumor was the intersect of both segmentations. If DSC^Tu^ was < 0.95, the two readers constructed the final tumor together.

The segmented removed tissue was then split into the curettage cavity and bone window plus tunnel, with the bone window plus tunnel being the removed bone until the last sagittal plane before the tumor was reached, and the curettage cavity being the rest. Remaining tumor was calculated by subtracting the curettage cavity from the tumor. Bone curettage was calculated by subtracting the tumor from the curettage cavity. Maximal remaining width was determined by calculating the Hausdorff distances between the tumor and curettage cavity surfaces, and selecting the largest Hausdorff distance, performed using the free and open source MeshLab software (www.meshlab.net) [12].

### Outcomes

The main outcome measure was accuracy of the procedure. Cartiaux et al. define surgical accuracy as “the closeness of agreement between an achieved surgical gesture and the desired surgical gesture” [13]. For this experiment, the desired surgical gesture was removing the entire tumor, which in terms of volume is the tumor volume. The achieved surgical gesture was the material that was removed, which in terms of volume is the curettage cavity. If accuracy is the closeness of agreement between the tumor and the curettage cavity, this can be quantified using a DSC, similarly to what was done during tumor segmentation: $DSC^{Ac} = \frac{{2\left| {Tumor\,\cap\,Curettage\,cavity} \right|}}{{\left| {Tumor} \right| + \left| {Curettage\,cavity} \right|}}$.

Descriptive statistics in the form of means and standard deviations calculated using Excel were used to report the remaining tumor, bone curettage, DSC^Ac^, maximal remaining width and procedure time for both procedure types (freehand/navigated) and user types (novice/expert). Within each user, the freehand and navigated groups were descriptively compared for any potential effect of navigation. This potential effect of navigation was then descriptively compared between the expert and novice user.

## Results

### Cadaveric bone tumor model

The bones varied in size and density. Smaller bones had no space for anteroposterior variation of tumor location. Bone density variation resulted in a variation of contrast between tumors and bones on the CBCT scans (Fig. 3a, b). All tumors were distinguishable from bone, but some more clearly than others.

As both tumor halves were created separately, there was some misalignment in tumor borders in either the craniocaudal or anteroposterior direction after the bones were glued back together. Based on a visual assessment of the CBCT scans, this misalignment was < 1 mm for 10/20 tumors, between 1–2 mm for 5/20 tumors, and between 2–3 mm for 5/20 tumors. Eight tumors had a small air cavity in their center, never wider than 2 mm, which was assumed to be a result of paraffin wax shrinkage. There were no further visible effects from paraffin wax shrinkage.

The resulting twenty tumors for the expert measurements, navigation and freehand groups combined, had a mean volume of 9.46 cm^3^ (SD 5.17). Twelve bones (2 proximal femurs, 6 distal femurs and 4 proximal tibias) were re-usable for the novice procedures. The eight bones that could not be used again after the expert experiments either showed a connection between the curettage cavity and medullary cavity on post-CBCT, making refilling impossible, or had a bone defect that would compromise the surgical approach. Filling the cavities gave a mean tumor volume of 15.96 cm^3^ (SD 5.08).

### Experimental setup

Nineteen expert procedures were executed as planned. One navigated procedure failed and was excluded because the two bone halves separated during curettage. The paraffin wax mixture approached the feeling of tumor material well, and as such the transition from tumor to bone could be used as a realistic feedback mechanism by the surgeons. The material was, however, more adhesive than tumor, so it tended to stick to the curette and the bone.

All novice procedures were executed as planned. The incurettable, hard and smooth material that was used to close the bone window from the first experiment felt notably different from cancellous bone. The proximal femur tumors were too large to be entirely removed due to the limited angular reach of the curette.

### Image analysis

A good fusion between pre- and post-CBCT and a good segmentation of the removed tissue was achieved in all cases. The two readers had a DSC^Tu^ > 0.95 for the tumor segmentations in 7/19 expert bones and 3/12 novice bones, with a total median DSC^Tu^ of 0.94 (IQR: 0.93–0.96).

The final mean tumor and curettage cavity segmentation volumes for all four treatment groups are specified in Table 1.

**Table 1:** Image analysis mean (standard deviation) segmentation volumes

	Expert (*n* = 19)		Novice (*n* = 12)	
	Navigated (*n* = 9)	Freehand (*n* = 10)	Navigated (*n* = 6)	Freehand (*n* = 6)
Volumes (cm^3^)				
Tumor	10.54 (6.72)	8.49 (2.85)	15.77 (5.74)	16.15 (4.32)
Curettage cavity	16.15 (6.02)	13.33 (5.81)	14.88 (4.15)	16.26 (3.84)

### Outcomes

**Fig. 4:**
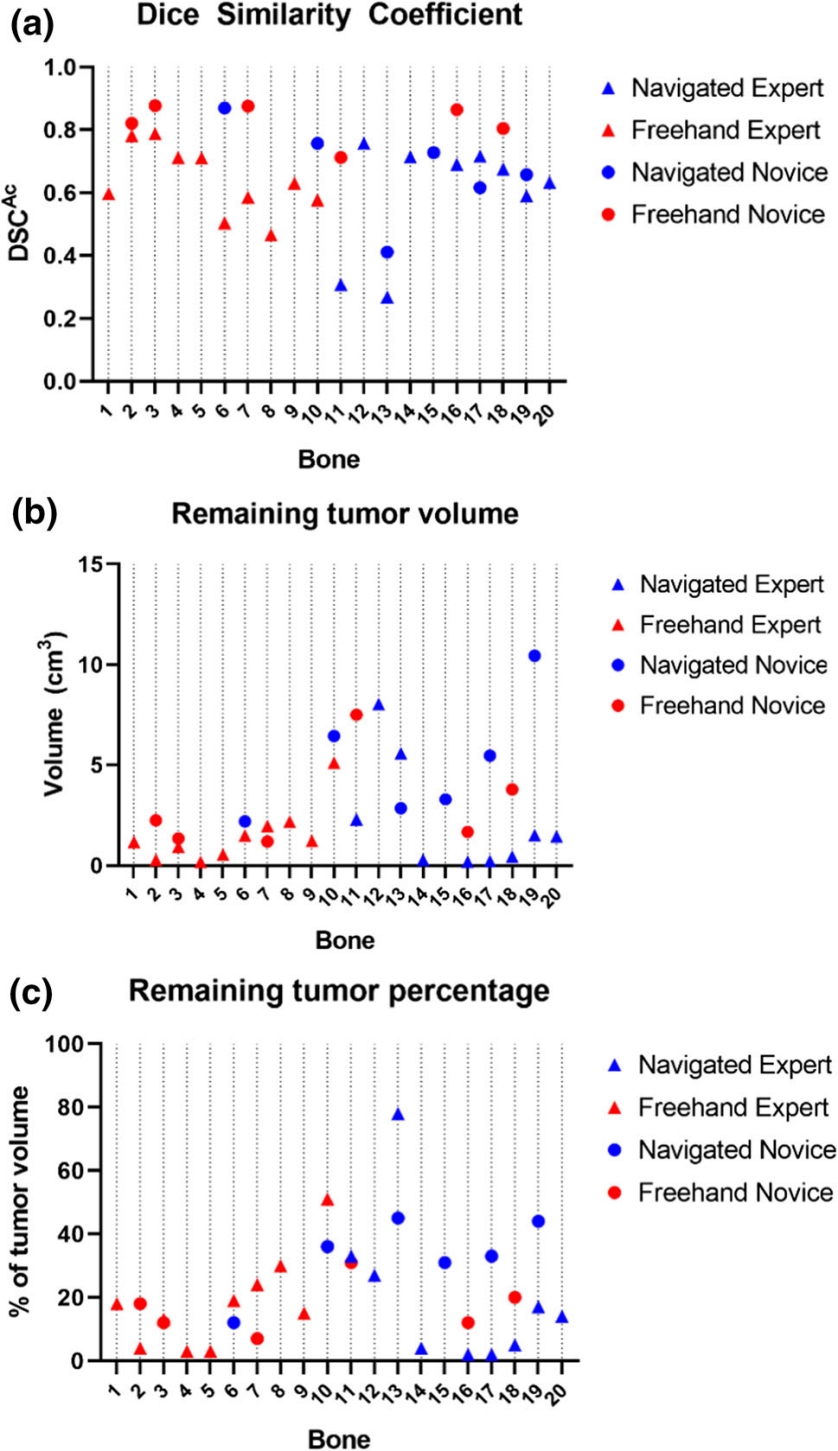
Graphs showing key outcomes for each individual bone in the navigated and freehand groups and the expert and novice experiments. The bone ID represents the code for each bone which was kept the same for both user experiments, it does not indicate the treatment order. Bone 15 was excluded from the expert experiments as the two bone halves separated during curettage, but it could be re-used for the novice experiments together with eleven other bones (bone 2, 3, 6, 7, 10, 11, 13, 15, 16, 17, 18, 19); **a** The accuracy using the Dice Similarity Coefficient (DSC^Ac^); **b** the absolute remaining tumor volume and **c** the relative remaining tumor volume as a percentage of the initial tumor volume

**Fig. 5:**
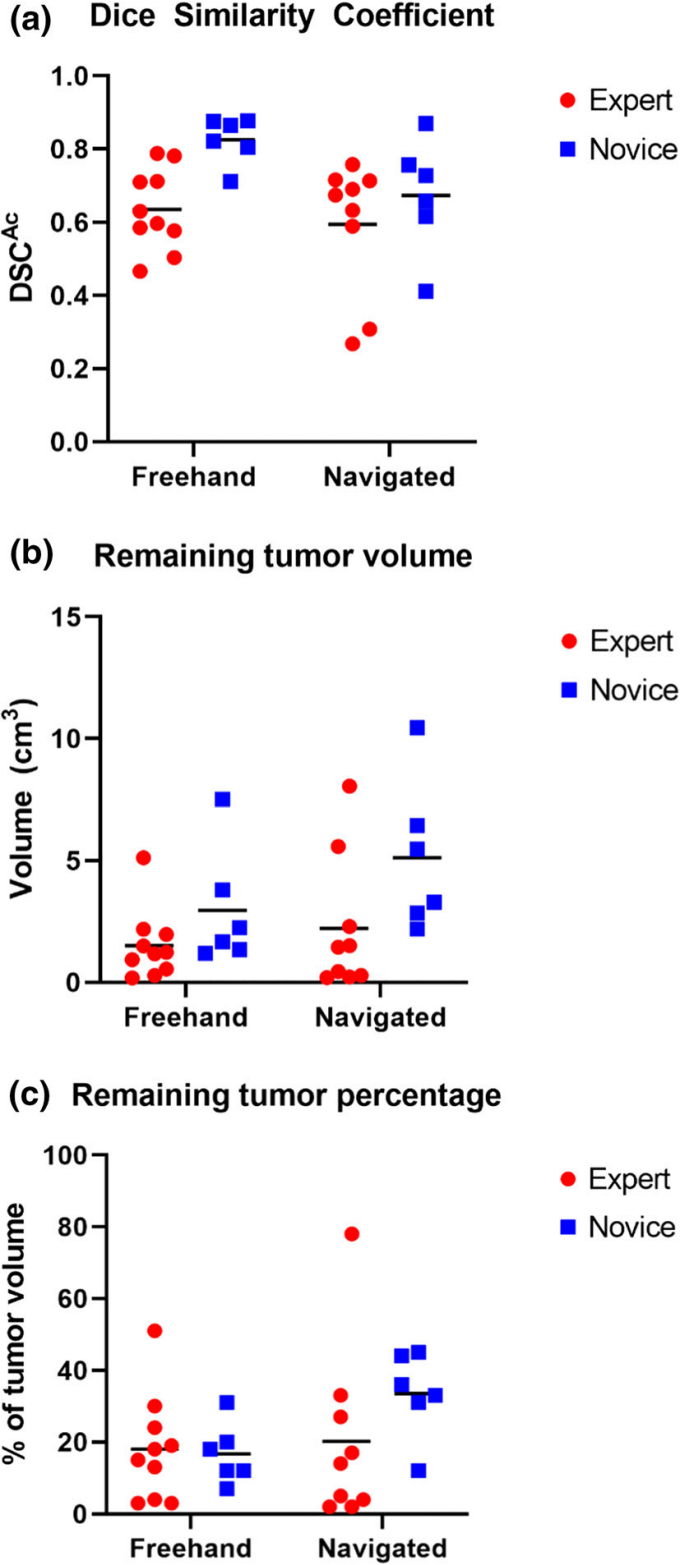
Grouped scatterplots illustrating the effect of navigation on key outcome values for the expert and novice user, with means indicated by the horizontal lines. **a** The accuracy using the Dice Similarity Coefficient (DSC^Ac^); **b** the absolute remaining tumor volume; and **c** the relative remaining tumor volume as a percentage of the initial tumor volume

The mean outcomes with a sample size of 19 for the expert user and 12 for the novice user are listed in Table 2. The effect size of navigation for the expert user seemed small, with outcomes being fairly similar for the navigation and freehand groups. The effect size of navigation for the novice user was favorable to the freehand group with a higher DSC^Ac^, less remaining tumor and bone curettage, and lower maximal positive margin. The DSC^Ac^ for each individual bone is shown in Fig. 4a, and the effect of navigation on DSC^Ac^ is visualized in Fig. 5a.

**Table 2:** Mean (standard deviation) study outcomes

	Expert (*n* = 19)		Novice (*n* = 12)	
	Navigated (*n* = 9)	Freehand (*n* = 10)	Navigated (*n* = 6)	Freehand (*n* = 6)
DSC^Ac^	0.59 (0.17)	0.64 (0.10)	0.67 (0.14)	0.83 (0.06)
Remaining tumor (cm^3^)	2.22 (2.61)	1.51 (1.36)	5.12 (2.81)	2.96 (2.21)
Bone curettage (cm^3^)	7.82 (4.16)	6.41 (3.43)	4.23 (2.75)	3.07 (1.48)
Max. remaining width (mm)	5.99 (3.75)	6.68 (2.77)	10.32 (3.40)	6.59 (2.29)
Time (s)	333 (92)	315 (86)	499 (37)	464 (58)

**Fig. 6:**
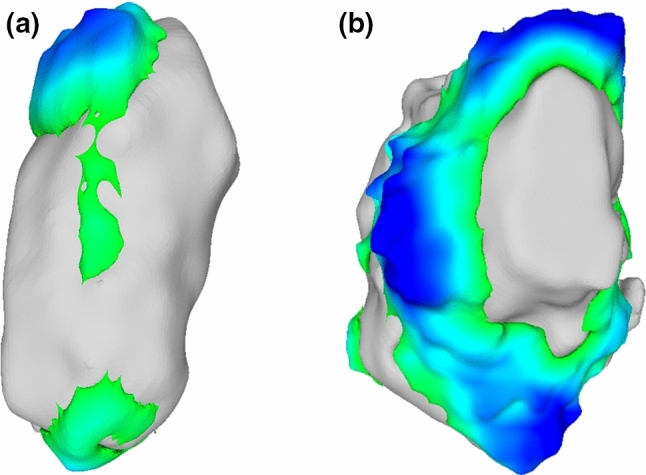
Visualizations of the remaining tumor in various bones; **a** Is a 3D model showing the remaining tumor in a few isolated spots in the expert bone 3. The curettage cavity is depicted in gray, and the remaining tumor is colorized, with green being a small width and dark blue being the maximal remaining tumor width of in this case 5.1 mm; **b** Illustrates the more diffusely spaced remaining tumor in the novice bone 11. The color range is the same as in (**a**)

All navigated and freehand procedures had some amount of remaining tumor, with means of 2.2 (2.6) cm^3^ (mean 20% of the tumor volume) and 1.5 (1.4) cm^3^ (18%), respectively, for the expert and 5.1 (2.8) cm^3^ (33%) and 3.0 (2.2) cm^3^ (17%), respectively, for the novice. The absolute and relative remaining tumor volumes for each individual bone are shown in Fig. 4b, c, and the effect of navigation these remaining tumor volumes is visualized in Fig. 5b, c. This remaining tumor was generally located on the edges in a few isolated spots for the expert user (Fig. 6a), and more diffusely spaced for the novice user (Fig. 6b). Most procedures (17/19 expert bones and 8/12 novice bones) were without strands of remaining tumor protruding > 5 mm to the center of the cavity. Although the expert user had four navigated and three freehand bones with only ≤ 5% remaining tumor, both groups also contained one bone with a large amount of remaining tumor (78% and 51% respectively).

The mean maximal remaining tumor width stood out for the navigated novice group with 10.3 (3.4) mm against 6.0 (3.8) mm for the navigated expert, 6.7 (2.8) mm for the freehand expert and 6.6 (2.3) mm for the freehand novice groups.

## Discussion

In this explorative study, there were little differences in curettage accuracy between a navigated and freehand approach on a cadaveric bone tumor model for an expert user, whereas a novice user was more accurate without navigation. The expert user showed a comparable relative curettage cavity size for both approaches, with means of 20% (navigation) and 18% (freehand) of the tumor volumes remaining in isolated spots on the edges of the cavity. The results from our study are not directly clinically translatable yet, because of differences between clinical and laboratory settings.

A few studies investigated the effectiveness of navigation to improve clinical outcomes of curettage. One retrospective study on curettage plus adjuvant phenol and ethanol of atypical cartilaginous tumors in long bones compared navigation to freehand. Their navigation setup was similar to ours, although they did segment the tumor before the procedure. Residual tumor was found in 2 out of 17 navigated patients, which was similar to the 7 out of 60 freehand patients that showed residual tumor [7]. The apparent lack of effect navigation had on the treatment outcome is consistent with our results. Lee et al. investigated the accuracy of navigated curettage with adjuvant navigated burring of the curettage cavity wall, which is a mechanical adjuvant therapy that extends the bony margin by a few millimeters [6]. They completely removed the tumor in all eight cases, based on a fusion of preoperative and postoperative CT. Navigated burring would seem an effective adjuvant to our curettage only results. However, Lee et al. used a wide cortical window to visualize the entire tumor for their straight burr, whereas we prefer a minimally invasive approach. They did propose that dedicated, curved instruments could reduce the cortical window.

According to Cartiaux et al., surgical accuracy depends on: (1) the level of assistance integrated into the procedure (navigation or freehand); (2) the user’s experience (expert or novice); and (3) local (anatomical) difficulties inherent to the procedure [13]. The expert and novice outcome volumes cannot be compared directly as the tumors were shaped, sized and approached differently (dependency 3) between the users. However, we examined the effect of navigation on accuracy within both user groups. Interestingly, navigation seems to have a negative effect on accuracy for the novice user. Both the remaining tumor and bone curettage were higher for the navigation group. This effect could be explained by the learning curve; lack of experience with navigation made the procedure more complicated and had a negative effect on the results. Furthermore, there was more tumor variation in the novice group as compared to the expert group due to the different way in which the tumors were created, and the novice group contained only 12 bones.

The difference in accuracy between the expert (DSC^Ac^ 0.59 [0.17] and 0.64 [0.10]) and novice user (DSC^Ac^ 0.67 [0.14] and 0.83 [0.06]) stems from different surgical techniques. The expert removed relatively more surrounding bone, thus lowering the DCS^Ac^, but also had slightly less remaining tumor. Although the expert was less accurate in terms of DCS^Ac^, it paints an incomplete picture of the success of the procedure because clinical curettage is only successful if the tumor does not recur. It seems that the expert was willing to sacrifice more healthy bone to achieve removing as much tumor as possible. Clinically, curettage is combined with adjuvant therapy because some (microscopic) amount of tumor always remains after an intralesional procedure. Adjuvant therapy increases the surgical margin, with the amount of increase depending its type [1]: phenol has a necrotic depth of up to 1 mm [14], whereas cryosurgery can reach up to 7–12 mm [15]. The mean maximal remaining width in this study was 6.4 mm (SD 3.3) for the expert user. Although the absolute number cannot be clinically translated, a certain amount of residual tumor can be the reason there are still tumor recurrences, even with adjuvant therapy. If the residual tumor after initial curettage was lower, overall therapy success would increase. Our results show there is room for improvement to the curettage procedure, especially when treating tumors with a higher recurrence rate.

This study is the first to investigate the accuracy of bone tumor curettage with and without navigation in a controlled setting and to image the curettage cavity, so the removed volume could be measured exactly. The cadaveric bone tumor model and specific outcome parameters that were proposed can be used by others for future studies, using free software for analysis to help the reproducibility. The laboratory setting allowed for a comparison between an expert and novice user, which cannot be done clinically.

This study also had its limitations. First, because this is an explorative study only 20 bones were used, which does not yield enough power for statistical significance, and the user groups only consisted of one user. The outcomes can, however, be used as a base for future studies. Second, cadaveric bones are mostly of a high age, which is only a partial representation of patient age. Consequently some bone densities were relatively low, giving low contrast to the tumor material on CBCT and making tumor segmentation more challenging, albeit all cases were still realistic. Tumors were not planned and segmented before the procedure as this is not our clinical practice. This did, however, mean there could be discrepancies in what surgeons and researchers thought to be tumor. Variability was reduced by having two readers. Third, paraffin wax and bone tumor might react differently to curettage. However, a similar tactile feedback was the most important characteristic for our research question. Any unwanted influence of phantom material properties on curettage would be equivalent for all procedures. Fourth, refilling the cavities left by the expert experiments for the novice experiments led to larger and differently shaped tumors than before, making an absolute comparison between expert and novice impossible. The objective of assessing the effect of navigation on the accuracy for both users was, however, attainable.

Surgical navigation is a useful image-guidance technology, but it is costly, especially in combination with intraoperative 3D imaging. These systems, and medical technology in general, should therefore only be used when there is an expected health benefit. Our previous study did report an added benefit of navigation to curettage in terms of more control in challenging locations (nearby vital structures) [5]. Cartiaux et al. described such a location as having a narrow safety zone, i.e., little tolerance in relation to the surgical target, so accurate curettage is required [13]. However, in that clinical study the safety zone was often only narrow in a small region or plane, whereas the present study measured accuracy around the entire tumor. Perhaps the current navigation setup, which is intended to use on bone but optimized for trauma and spine applications such as screw placement, visualizes a small region of interest that is limited to a few planes better than a large region that is focally and spherically distributed like the entire tumor border. The desired surgical gesture also differs between, e.g., pedicle screw placement, the accuracy of which benefits from navigation [16], and curettage. Some surgical gestures probably benefit more from navigation than others, but the navigation setup for curettage has room for improvement and more tailored software and hardware for curettage might help to reduce residual tumor rates and thus improve surgical accuracy in the future. Not all tumor types and procedures require improvement though, as the addition of adjuvant therapy generally already gives good clinical outcomes. It would be interesting to reproduce these results in complex clinical cases with a high recurrence rate.

In an explorative study on 20 cadaveric bone tumor models, navigated curettage in its current setup does not seem to be more accurate than freehand curettage. Still, the amount of remaining tumor showed that curettage has room for optimization. The novice user was less accurate using navigation as compared to freehand. Additionally, the novice user removed less healthy bone compared to the expert user, but thereby also had more remaining tumor.

## References

[1] Martinez M, Hwang J, Beebe KS (2014). Local adjuvants for benign aggressive bone tumors. Curr Orthop Pract.

[2] Jeys L, Matharu GS, Nandra RS, Grimer RJ (2013). Can computer navigation-assisted surgery reduce the risk of an intralesional margin and reduce the rate of local recurrence in patients with a tumour of the pelvis or sacrum?. Bone Jt J.

[3] Cartiaux O, Banse X, Paul L, Francq BG, Aubin C-É, Docquier P-L (2013). Computer-assisted planning and navigation improves cutting accuracy during simulated bone tumor surgery of the pelvis. Comput Aided Surg.

[4] Bosma SE, Wong KC, Paul L, Gerbers JG, Jutte PC (2018). A cadaveric comparative study on the surgical accuracy of freehand, computer navigation, and patient-specific instruments in joint-preserving bone tumor resections. Sarcoma.

[5] van Steenbergen TRF, van der Geest ICM, Janssen D, Rovers MM, Fütterer JJ (2019). Feasibility study of intraoperative cone-beam CT navigation for benign bone tumour surgery. Int J Med Robot Comput Assist Surg.

[6] Lee H-I, Shim JS, Jin HJ, Seo SW (2012). Accuracy and limitations of computer-guided curettage of benign bone tumors. Comput Aided Surg.

[7] Gerbers JG, Dierselhuis EF, Stevens M, Ploegmakers JJW, Bulstra SK, Jutte PC (2018). Computer-assisted surgery compared to fluoroscopy in curettage of atypical cartilaginous tumors / chondrosarcoma grade 1 in the long bones. PLoS ONE.

[8] Bauer HCF, Brosjö O, Kreicbergs A, Lindholm J (1995). Low risk of recurrence of enchondroma and low-grade chondrosarcoma in extremities:80 patients followed for 2–25 years. Acta Orthop.

[9] Di Giorgio L, Touloupakis G, Vitullo F, Sodano L, Mastantuono M, Villani C (2011). Intralesional curettage, with phenol and cement as adjuvants, for low-grade intramedullary chondrosarcoma of the long bones. Acta Orthop Belg.

[10] Kim W, Lee JS, Chung HW (2018). Outcomes after extensive manual curettage and limited burring for atypical cartilaginous tumour of long bone. Bone Jt J.

[11] Kikinis R, Pieper SD, Vosburgh KG, Jolesz FA (2014). 3D slicer: a platform for subject-specific image analysis, visualization, and clinical support. Intraoperative imaging and image-guided therapy.

[12] Cignoni P, Rocchini C, Scopigno R (1998). Metro: measuring error on simplified surfaces. Comput Graph Forum.

[13] Cartiaux O, Jenny J-Y, Joskowicz L (2017). Accuracy of computer-aided techniques in orthopaedic surgery. J Bone Jt Surg.

[14] Lack W, Lang S, Brand G (1994). Necrotizing effect of phenol on normal tissues and on tumors: a study on postoperative and cadaver specimens. Acta Orthop.

[15] Malawer MM, Marks MR, McChesney D, Piasio M, Gunther SF, Schmookler BM (1988). The effect of cryosurgery and polymethylmethacrylate in dogs with experimental bone defects comparable to tumour defects. Clin Orthop Relat Res.

[16] Mason A, Paulsen R, Babuska JM, Rajpal S, Burneikiene S, Nelson EL, Villavicencio AT (2014). The accuracy of pedicle screw placement using intraoperative image guidance systems: a systematic review. J Neurosurg Spine.

